# Believing and the disposal of bodies after death

**DOI:** 10.3389/fpsyg.2022.971284

**Published:** 2022-10-04

**Authors:** Courtney Applewhite

**Affiliations:** University of California, Santa Barbara, Santa Barbara, CA, United States

**Keywords:** death and dying, meaning making process, death, global meaning, non-religious people, secular people, ritual, promiscuous teleology

## Introduction

One of the first realities of death is the presence of the physical dead body. In studies of death in fields such as neuroscience or psychology, we focus on the biological basis or clinical implications of grief and bereavement (Neimeyer, 2004). But grief and bereavement are a response to death and, thus, a dead body. Psychologists have paid little attention to the material body of the deceased and the meaning-making processes associated with it (White et al., 2016). I will approach the meaning making process from two perspectives: the first, how our beliefs affect the appraisal of the deceased body of another person, perhaps a loved one, and the second, how those same beliefs may be similar or different in how we appraise our own deceased body as we consider what we want done with it after our own death.

I aim to shift the focus from the experience of grief specifically to the construction of meaning in relation to the physical body after death or, as they say in the profession, the choice of body disposition. To do so, we can turn to Park (2010) integrated notion for meaning making as it relates to how people might appraise a dead body. While cremation and conventional burial practices (in which a person is embalmed, placed in a casket, and then buried in a vaulted grave) are still the most popular choices for bodily disposition in the United States, other practices like green or natural burial, alkaline hydrolysis, and natural organic reduction are becoming more widely available and requested, particularly by non-religious people. I argue that in choosing these alternative methods of bodily disposition for themselves or for their loved ones, non-religious people are enacting a different kind of belief by simultaneously recognizing the materiality of the body and ascribing value and meaning to it from spiritual, environmental, and/or cultural perspectives. This way of viewing appraisal will draw from a relational-deictic framework and consider how people often hold simultaneous and sometimes contradictory appraisals.

## Integrated meaning making and global meaning

Our global meaning systems are made up of beliefs, goals, and subjective feelings as well as internal representations of desired processes, events, or outcomes (Park, 2010). These beliefs, which take many forms, e.g., religious, spiritual, ethical, or material, guide how we make sense of the world around us. As we encounter stressful moments throughout life, we must make sense of them in some way; we must ascribe meaning to them. In forming meaning in these moments, called situational meaning, we must attempt to either assimilate–change our view of the situation–or accommodate–change our global meaning. This process is not simply cognitive, but also relies on emotional processing. For many, viewing a dead body and/or making decisions about how to dispose of it is one of those stressful moments.

The meaning-making process begins with the physical reality of the deceased body. There are two separate ways to approach the idea of the deceased body. One is through the experience people have in choosing a disposition method for another person at the time of their death. Another is making a future appraisal of your own dead body. Keeping these two kinds of events in mind, we can consider how non-religious individuals in the United States react to death when they often lack the institutionalized rituals and routines for responding to a death that religious institutions typically provide (MacMurray and Fazzino, 2017). For example, if you are Catholic in the United States, most of the time from the guidance of your parish priest you would choose to bury a loved one or to personally be buried in a Catholic cemetery. There are rituals and masses associated with death that are congruent with your global meaning structure. The growing number of people in the United States that do not subscribe to any religious tradition do not have the rituals, rites, and ceremonies around bodily disposition and therefore do not necessarily move as smoothly through the meaning making process when deciding what to do with the deceased (Kosmin et al., 2009; MacMurray and Fazzino, 2017; Smith and Cragun, 2019). As such, they must find new ways to make meaning that can help them make sense of death and dispose of the body in a way that makes sense to them *as secular people*.

## Shifting disposition methods in the United States

The self-understanding that people brought to the dead used to be relatively consistent. In the antebellum United States, funeral services were presided over by Christian clergy, occurred soon after death, and were rarely attended by anyone other than immediate family (Laderman, 1996). The family's concerns centered on religious beliefs, namely the deceased's soul and whether it was bound for heaven. Both the form and content of these services shifted after the Civil War. After the 1860s, technological advancement allowed for the professionalization of the funeral industry, notions about the ontology of heaven and hell changed, and there was extended time before burial (Laderman, 1996; Prothero, 2001). Together, this meant that the presentation of an embalmed corpse in an open casket in the context of a religious service became popular in the United States and, until about the 1960s, was the norm.

Cremation increased in popularity in the 1960s in response to Jessica Mitford's aggressive critique around pricing in the funeral industry, the Catholic Church lifting the cremation ban, and the rise of the counterculture (Prothero, 2001). But cremation numbers remained relatively low even in the 1990s. Since 2005, the Cremation Association of North America has been collecting data on cremation rates based on their members and affiliates. Their data demonstrate that cremations have been climbing steadily only to surpass the number of burials in 2015 and come to represent more than 50% of dispositions in 2016. Cremation rates have been increasing steadily over the past several decades; there has not been an overwhelming jump or shift since that period in the 1960s (Kemmis, 2021). This trend is like much of Europe, although the numbers in Europe have increased to even greater heights in many places. Cremation is not the only “new” practice growing in popularity. Other, more diverse options are also arising.

## Non-religious people and disposition choice

Interest in alternative forms of disposition is on the rise (National Funeral Directors Association, 2022). The National Funeral Directors Association found that over 60% of people expressed interest in green burial in particular. This is also demonstrated by the increase in the availability of alternatives to cremation and conventional burial like alkaline hydrolysis, or water cremation, and natural organic reduction (i.e., human composting) as well as body donation, which has long been favored by the non-religious–specifically Atheists (Copeman and Quack, 2015). As the numbers of non-religious people rise in the United States, there may be greater opening to exploring utilitarian or other disposition options that do not have religious associations (Marsh, 2021). One of the values that may be applied to disposition is a concern for the environment (van Mulukom et al., 2022). Even though there is an important overlap in environmental protection impulses in both religious and non-religious individuals, in death these innovative and “green” practices are relatively free of religious baggage (Beaman, 2017).

I suggest that the focus on the environmental impact of their own deceased body indicates an appraisal of that body as less sacred and more material. It may also demonstrate how secular people are creating practices for themselves around death that reflect a change in belief. Non-religious people often do not believe that the body is critical to any kind of afterlife, so they are reframing the appraisal. But their intentional choices suggest that non-religious people are actively seeking actions associated with their understanding of death and the dead body that align with their values and beliefs in life, even those not religion related.

## How belief influences disposition: Relational-deictic framework

Alongside a change in disposition preference, we are also seeing an increasing number of people who do not believe in a particular afterlife. One-in-six Americans do not believe in any afterlife at all (Pew, 2021). When discussing non-religious individuals, I am describing a subsection of this identification that have a materialist worldview and are part of this group that does not believe in a particular afterlife. This certainly does not describe the plurality of people who identify as non-religious. But within this group, we see this belief pattern that has an increasing number of people believing that death is the final end and wanting these environmentally friendly disposition practices.

To further consider how non-religious people might be thinking about the dead body, I propose that we turn to a “relational-deictic” interpretation of the physical world. Appraisal through a relational-deictic interpretation is an alternative to promiscuous teleology or the bias toward purpose-based reasoning (ojalehto et al., 2013). Teleological explanations become promiscuous when applied to natural objects rather than artifacts. For example, the clouds exist because they provide shade. These biases exist in humans from childhood into adulthood, although often not explicitly, and religion can be thought of as a product of this kind of reasoning (Kelemen et al., 2013). In this example, clouds would be a product of divine creation. As an alternative, the relational-deictic framework takes into account the importance of relational and ecological reasoning as well as the points of view within that relation (ojalehto et al., 2013). Rather than taking an intentional design stance with respect to natural forms, which presupposes that the purpose arises from a designer or a sole source, the relational-deictic stance assumes that purposes come from multiple sources and therefore purpose arises from the perceiver's *sense* of purpose. This becomes critical when considering the reality of the dead body.

While ojalehto et al. (2013) point out that it is Indigenous populations in the United States that most clearly display this kind of cognitive approach, I argue that this reasoning can be a better way to talk about the cognition of the non-religious people who are choosing alternative disposition methods. The key relationship is between the living and the dead body. From this relationship emerges additional connections because both are part of the natural world, but one will continue on existing in that natural world (the living) and another will decompose. So, although the living recognizes the dead body as part of that cycle of nature, as material, it remains important because of the ongoing connection with the person the body used to be. From a teleological perspective, death may be “part of God's plan,” or it may “happen for a reason,” but these phrases are not only laden with religious connotation, but also fundamentally incongruent with many non-religious people who subscribe to materialist worldviews. And yet, even if you do not believe in an afterlife, or believe in a soul, or the sacrality of the body from a religious or spiritual perspective, which many non-religious people do not, you still maintain a relationship to that person who has died. When we appraise the deceased body of another person, particularly a loved one, we see that body as more than simply material.

Using a relational-deictic framework would shift the language associated with death, particularly when choosing these alternative forms of disposition, to phrases like, “the body is part of a natural cycle,” or “the person's body will go back to the earth to support it.” Hence, the body becomes a key part of a purpose or cycle without the baggage of promiscuous teleology. This framework is critical to the non-religious because they lack theistic global meaning about the purpose of the dead body, or death in general, and the traditional forms of disposition do not necessarily allow the same kind of reasoning. Conventional burial and cremation do not lend themselves to this natural return to the earth as easily.

## Discussion

Relational frameworks use sophisticated ecological reasoning that is particular to Indigenous communities, but this argument suggests a biological basis to this process, which may be why we apply it in considering the cognitive underpinnings of non-religious people. And a possible pathway for this cognitive logic is that there is a coexistence of natural and supernatural explanations for things within people's minds for both themselves and for others (Legare and Shtulman, 2018). The physical body can represent the vessel for the soul, but it is also a biological fact. The physical body may both be important and not be important. Relational frameworks are most important for the bodies of those to whom people are related. This explains why some people tend to be quite flippant about their own death and their own bodies after death but would very rarely be as cavalier about a loved one's body. This suggests that global meaning is not necessarily a fixed or total system, but it can have contradictory pieces that are held simultaneously and constantly shifting.

Global meaning systems can consider several different causalities and with several contradictory points of view within the same person, but then we return to the physical reality of the dead body. This short article suggests that as non-religious people are moving away from the dominant religious narratives that provide meaning and structure around the dead body for both themselves and others, they are introducing other kinds of meaning. These meanings include values and beliefs around environmentalism, secularism, economics, or tradition outside of religion, which has perhaps influenced the growing numbers of people who are interested in green burial, natural organic reduction, and other means of bodily disposition. For them, the body is not sacred in the religious sense, but is indeed value-laden from a relational and natural perspective. Many, despite their non-belief, still ascribe a specialness to the deceased body, a cognitive and emotional response that bears further investigation. Future research may directly investigate how non-religious people think about the deceased body and relate to other phenomena around death, not limited to after-death communications, or sensing presences, and the experiences of grief.

## Author contributions

The author confirms being the sole contributor of this work and has approved it for publication.

## Funding

This paper was funded by Dr. Rüdiger Seitz, *via* the Volkswagen Foundation, Siemens Healthineers, and the Betz Foundation.

## Conflict of interest

The author declares that the research was conducted in the absence of any commercial or financial relationships that could be construed as a potential conflict of interest.

## Publisher's note

All claims expressed in this article are solely those of the authors and do not necessarily represent those of their affiliated organizations, or those of the publisher, the editors and the reviewers. Any product that may be evaluated in this article, or claim that may be made by its manufacturer, is not guaranteed or endorsed by the publisher.
